# Supplementary material for: Functional differences between TSHR alleles associate with variation in spawning season in Atlantic herring
Source: Commun Biol. 2021 Jun 25;4:795. doi: 10.1038/s42003-021-02307-7 (PMC8233318; doi:10.1038/s42003-021-02307-7)
Supplement: Supplementary file 2 — Supplementary Information [file 42003_2021_2307_MOESM2_ESM.pdf]

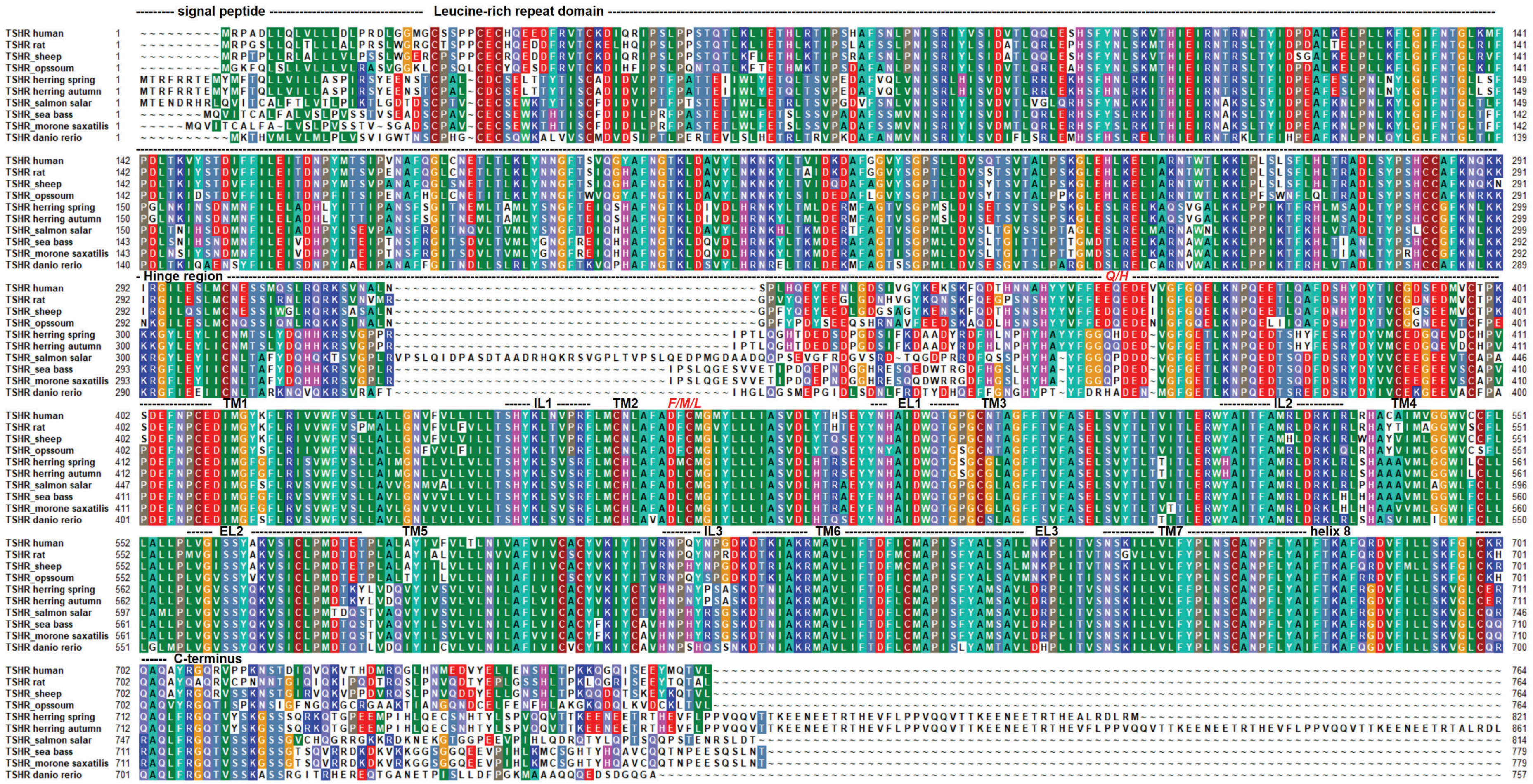

Supplementary Figure 1. Protein alignment among TSHR orthologs. Residues with hydrophobic (green), positively charged (blue) or negatively charged (red) side chains are highlighted. Predicted dimensions as parts of the receptor including LRRD, hinge region, TMH1 - 7, IL 1 - 3, EL 1 - 3, helix 8 and C - terminus are indicated.

a

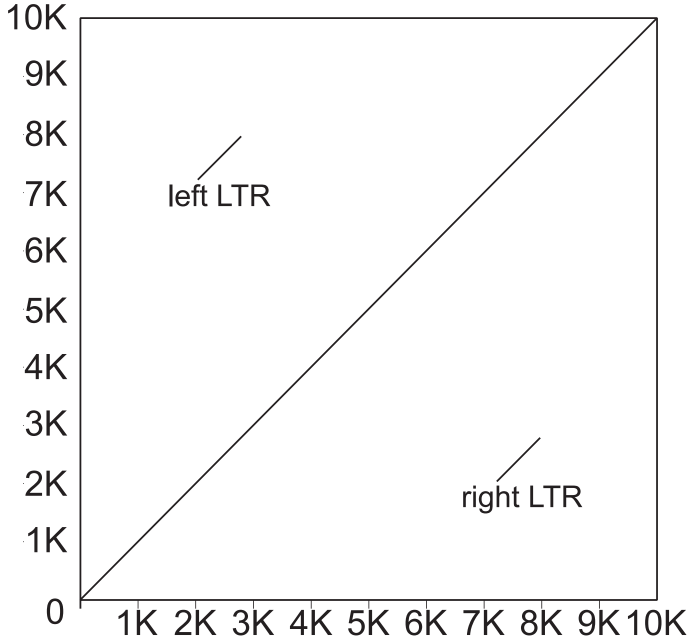

b

```
         1                                                                                                           100
 S-left  TGTGTAGGAG CCGCATTTAA GTTTTTTGTA TTTGGAATGA TTTATCGATT TTTCCCCTTT TTGTGGAATT GCGAGTGTTC GCCCTCTATA GGTGGTGACG
S-right  .......... .......... .......... .......... .....T.... .......... .......... ........G. .........C ..........
 A-solo  .......... .......... .......... .......... A....T.... .......... .......... ........G. .........C ..........
Pacific  .......... .......... .......... .......... .....T.... .......... .......... ........G. .......... ..........

         101                                                                                                         200
 S-left  GTGCCACCCT TGAAATGGCT GCCAGACACG CATGACGGGG AGAAGGCGGG CGCAATTACC GTCATTGACC TCAGTGCTGA GGCCGTGAAC CACGCTGTCT
S-right  .......... .......... .......... .......... .......... .......... .......... .......... .......... ..........
 A-solo  .......... .......... .......... .......... .......... .......... .......... .......... .......... ..........
Pacific  .......... .......... .......... .......... .......... .......... .......... .......... .......... ..........

         201                                                                                                         300
 S-left  CCGTTTGGGC CTGTTCAGCT TGTTGCAGCG TTTAGTTATT TGGACTTTAA AGCTGCAATT GCATCCTTAT TTTTTGTGAC AATTTTTCTA CAATAAAACC
S-right  .......... .......... .......... .......... .......... .......... .......... .......... .......... ..........
 A-solo  .......... .......... .......... .......... .......... .......... .......... .......... .......... ..........
Pacific  .......... .......... .......... .......... .......... .......... .......... .......... .......... ..........

         301                                                                                                         400
 S-left  GGCAACATTG GATGGTCAAA CTCCCTTTGT ATTGGATTAT TGGACAGAAC TGCGAGTCTT GACATTGCTT CTCCTGCTTT CTCCATGGTG GTAAAATCAA
S-right  .......... .......... .......... .......... .......... .......... .......... .......... ......A... ..........
 A-solo  .......... .......... .......... .......... .......... .......... .......... .......... ......A... ..........
Pacific  .......... .......... .......... .......... .......... .......... .......... .......... .......... ..........

         401                                                                     471
 S-left  TTAGGACGTA GGAATAGGAT CTAGGGGTTT AAAGGAAAAA ACTCAACTGT TTTTGCCACT ACAATTCGTC A
S-right  .......... .......... .......... .......... .......... .......... .........
 A-solo  .......... .......... .......... .......... .......... .......... .........
Pacific  .......... .......... .......... .......... .......... .......... .........
```

Supplementary Figure 2. Identification and analysis of BEL/Pao LTRs. (a) Dot plot analysis revealed two LTRs flanking the 5.2 kb structural variant in the spring allele. (b) Sequence alignment among the two LTRs of Atlantic spring allele (s-left and s-right), solo LTR of autumn allele (a-solo) and LTR of Pacific herring (Pacific-LTR). Four fixed differences between two spring LTRs are highlighed by red box and one unique difference fixed in the autumn allele is highlighed by green box.

a

Spring TSHR

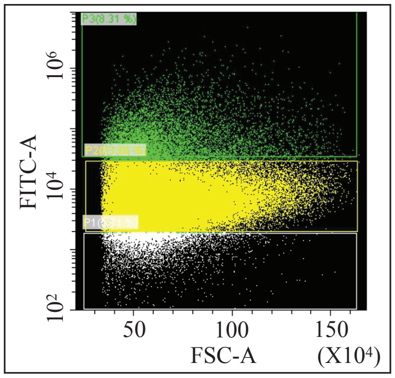

Population	Events	%Parent	Mean FITC-A
○ P1	5895	5.71%	1351.4
● P2	86704	83.98%	7842.6
● P3	8576	8.31%	109406.2

b

Autumn TSHR

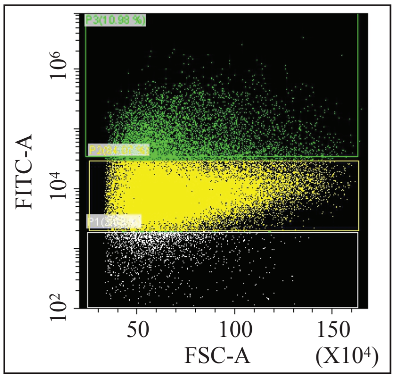

Population	Events	%Parent	Mean FITC-A
○ P1	1728	3.03%	1099
● P2	48019	84.07%	9547.6
● P3	6270	10.98%	109830.4

c

pcDNA3.1

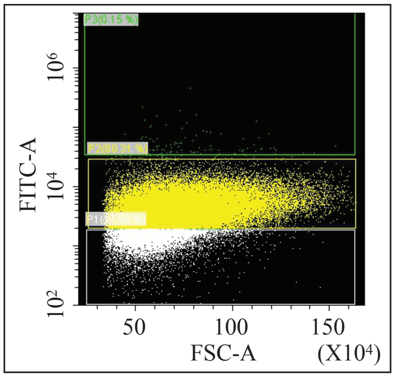

Population	Events	%Parent	Mean FITC-A
○ P1	14125	17.56%	1435.5
● P2	64617	80.31%	4815.7
● P3	122	0.15%	58510.3

d

Negative control

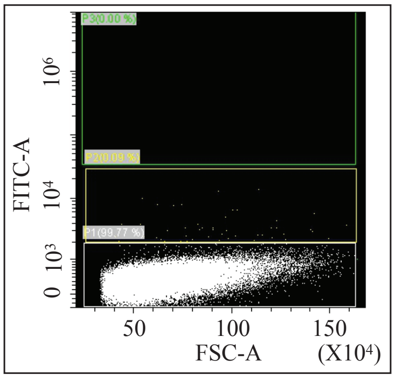

Population	Events	%Parent	Mean FITC-A
○ P1	56196	99.77%	388.2
● P2	50	0.09%	3979
● P3	0	0.00%	NA

Supplementary Figure 3. Cell surface expression of herring *TSHRs* measured by flow cytometry. (a) Spring TSHR. (b) Autumn TSHR. (c) Empty pcDNA3.1. (d) Negative control.

```
-720  ATAACACAAT GTTGTTTTGT AATTACAAGT AATCTACCAT GGAAACTTAT GACGGTGGTT

-660  GGCATCTGCC ACGACATGTT TATGACATGG GTTTATTAGT GTTTTGACAG AGAGTTCAAG

-600  TGAAGTGTCT CCCTCGTGGT ACATAACGCC TGGTGCTTTA CAAGAAGATC ATGACCTGTT

-540  TCAACGCATC CGCCTCCAGG AAAGGCTGAG GTTGGAAGAC TGCTCGCACG CTTTTGGCGT

-480  GAGCCAGGCG CTTTGTGGGC TCCTCTTCAG GCAGCCTCAT ACCATCTCAC CGCGATTTCC

-420  TCCACTTGAT GTAGTGCGTG CTCAGACAGC ACCGAAAGTT GTTTACCCTG CTTTAAAAAC

-360  ACTGGAAGTA ATTCCCATTT TCCTTCGTCA TAGTGGATGA TGTTGTAAAC ATTAGAGGTG

-300  AAGACAGCAC CTTTGACGTC AACTAGATGT TTCCGTTGAA GTATTACTAA TCCTGTCTGC
                 CRE element
-240  ATGTACACAA TGCAAGCAAA AGTGTAATCC CTGTATCTCA CTTAGTCTAA TTAAAAAACT

-180  GCGTCCGTAA TTACTAACCG GCATTTCTGT GTCTTCTTGG ATGATATCAG ATTCCCCGCT

-120  GTCCCTTTTC CATCAATTCA GTTTCCTTGT CAGTCCGGAG AAAAAGCCCA TAGGTGACGT
                                                              CRE element
 -60  CACGATCTCC CCACAAGATA AGCCCGAGAT TTATTTCAGC GCGGGGAATG ATTTACGGTA DIO2 TSS
```

Supplementary Figure 4. Genomic sequence upstream of the transcription start site (TSS) of *DIO2* in Atlantic herring. Two full cAMP response elements (CRE) around - 60 bp and - 290 bp are highlighed by yellow.

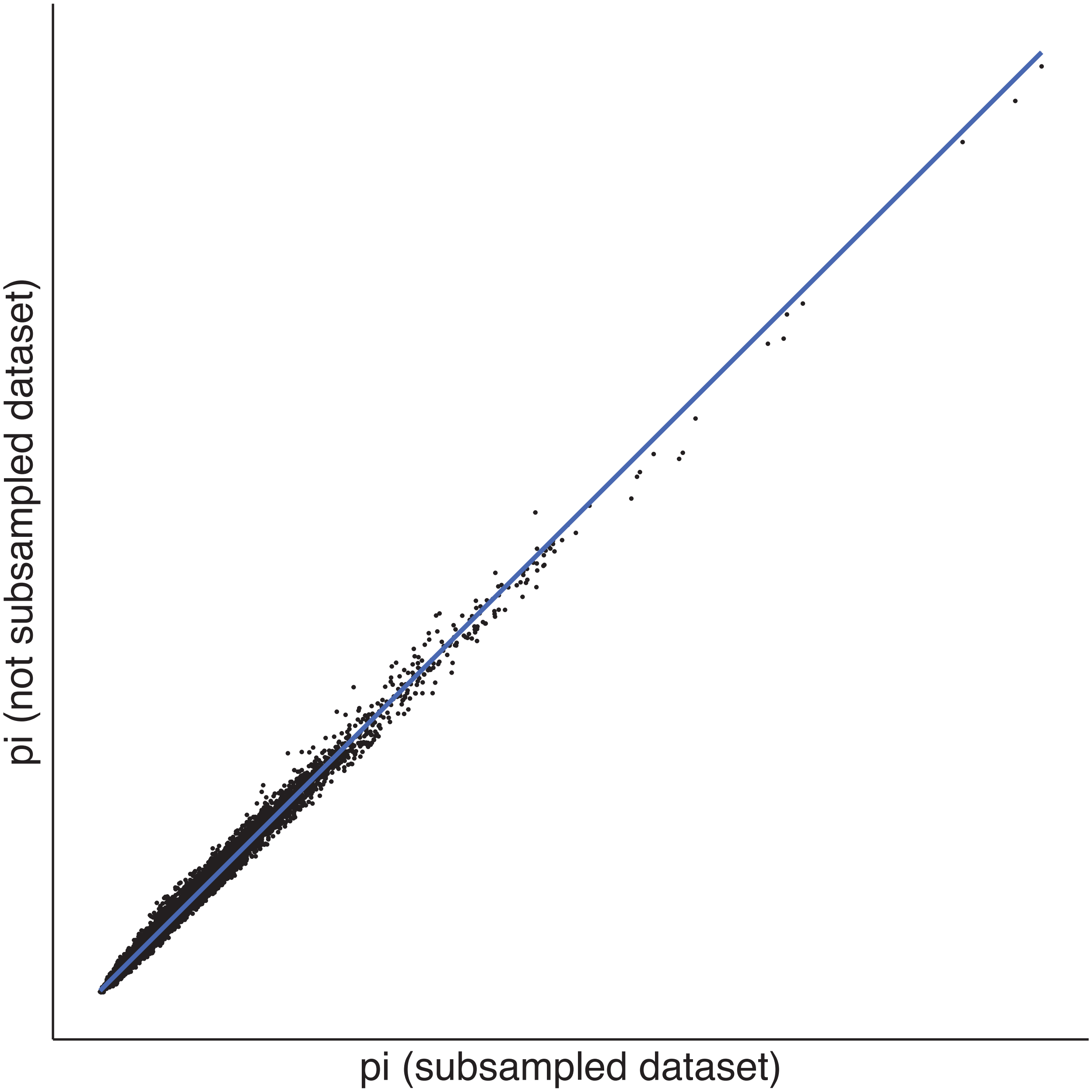

Supplementary Figure 5. Linear regression between pi values calculated from the coverage subsampled and not subsampled SNP datasets.

Supplementary Table 1. Primer and probe information.

Primer name	Forward (5' to 3')	TaqMan probe (5' to 3')	Reverse (5' to 3')	Utilization
5.2kb-confirm	GCACAGAGGCTGTTCTTCACTT		TCATGTTTTTGCATGTTCCATT	confirm the 5.2kb structural variant
geno22aa	AAGAGATGCCCATCCACTTG		AGTTAGGGAATGGAAAACATCG	genotype the 22aa repeat
geno5.2kb	1F: GTCTGAAGAGTGACCAGGGTTC		TGTTAACCCTTGGTTCACTGTC	genotype the 5.2kb structural variant
	2F: CGTGTCATCTGCAAATTTGATT			
DIO2-Outer	GCTGATGGCGATGAATGAACACTG		ACTAGCAACACCGAGTCGTACA	Outer 5' RLM-RACE PCR
DIO2-Inner	CGCGGATCCGAACACTGCGTTTGCTGGCTTTGATG		GCAGTATCTGCAAAGTCACCAA	Inner 5' RLM-RACE PCR
qTSHR	CACTACAATTCCAGCCAACTCA	CACGCTTTCAACGGGACCAAAC	TCTCGTCCAGCATCGTTAGATA	Tissue expression profile
qTSHB	GTTTCTCACTGCTGTGCTGCT		CCAGCTCTTTCAAGTTGCTGTC	Tissue expression profile
qDIO2	TGCGCTCTATATGGAACAGCTT		GACTCGAAGTCGAGGAGGTG	Tissue expression profile
qACTIN	CACCATTGGAAACGAGAGGT	TCTTCCAGCCTTCCTTCCTGGG	GTGTTGGCGTACAGGTCCTT	Tissue expression profile

Supplementary Table 2. Information associated to the Pool-seq data from Han et al.[16] that was used to perform a genome scan and to calculate nucleotide diversity (π) and Tajima's D per pool to characterize the selective sweep in the *TSHR* locus.

Sample name	Location	Latitude	Longitude	Spawning season	Sample size	5th coverage percentile[a]	99th coverage percentile[b]
PB5_Gävle_Baltic_Spring	Gävle	60.43	17.18	Spring	100	13	72
PB1_Hästskär_Baltic_Spring	Hästskär	60.35	17.48	Spring	50	13	80
Q_Norway_Atlantic_Atlantic_Spring	Norway	64.52	10.15	Spring	49	39	194
HGS15_NSSH_Atlantic_Spring	Norway	67.46	9.47	Spring	43	25	102
PB2_Iceland_Atlantic_Spring	Iceland, Höfn	65.49	-12.58	Spring	100	16	92
DalInB_Atlantic_Spring	Baie Des Chaleurs	48.00	-65.51	Spring	41	11	54
DalNsS_Atlantic_Spring	Northumberland Strait	46.19	-64.09	Spring	49	20	100
H_Fehmarn_Baltic_Autumn	Fehmarn	54.50	11.30	Autumn	50	22	122
PB7_Gävle_Baltic_Autumn	Gävle	60.44	17.35	Autumn	100	14	83
DalBoB_Atlantic_Autumn	Bonavista Bay	48.49	-53.20	Autumn	49	23	118
DalGeB_Atlantic_Autumn	German Banks	43.16	-66.18	Autumn	48	17	91
DalNsF_Atlantic_Autumn	Northumberland Strait	45.44	-62.36	Autumn	50	12	63
N_NorthSea_Atlantic_Autumn	North Sea	58.06	6.10	Autumn	49	26	138
HGS17_IsleOfMan_IrishSea_Autumn	Isle of Man	54.06	-4.37	Autumn	50	21	106

[a] It refers to the 5th percentile of the coverage frequency distribution of SNPs. This value was used as the minimum coverage threshold of SNPs included in the calculation of π and Tajima's D.

[b] It refers to the 99th percentile of the coverage frequency distribution of SNPs. This value was used as the maximum coverage threshold of SNPs included in the calculation of π and Tajima's D.
