# Supplementary material for: Functional differences between TSHR alleles associate with variation in spawning season in Atlantic herring
Source: Commun Biol. 2021 Jun 25;4:795. doi: 10.1038/s42003-021-02307-7 (PMC8233318; doi:10.1038/s42003-021-02307-7)
Supplement: Supplementary file 3 — Description of Supplementary Files [file 42003_2021_2307_MOESM3_ESM.pdf]

Description of Additional Supplementary Files

File name: Supplementary Data 1

Description: Raw data supporting the findings of this study.
