# Supplementary material for: Functional differences between TSHR alleles associate with variation in spawning season in Atlantic herring
Source: Commun Biol. 2021 Jun 25;4:795. doi: 10.1038/s42003-021-02307-7 (PMC8233318; doi:10.1038/s42003-021-02307-7)
Supplement: Supplementary file 5 — Reporting Summary [file 42003_2021_2307_MOESM5_ESM.pdf]

nature research

Corresponding author(s): Junfeng Chen
Leif Andersson

Last updated by author(s): Apr 29, 2021

Reporting Summary

Nature Research wishes to improve the reproducibility of the work that we publish. This form provides structure for consistency and transparency in reporting. For further information on Nature Research policies, see our Editorial Policies and the Editorial Policy Checklist.

Statistics

For all statistical analyses, confirm that the following items are present in the figure legend, table legend, main text, or Methods section.

n/a	Confirmed	
☐	☒	The exact sample size (*n*) for each experimental group/condition, given as a discrete number and unit of measurement
☒	☐	A statement on whether measurements were taken from distinct samples or whether the same sample was measured repeatedly
☐	☒	The statistical test(s) used AND whether they are one- or two-sided<br>*Only common tests should be described solely by name; describe more complex techniques in the Methods section.*
☒	☐	A description of all covariates tested
☒	☐	A description of any assumptions or corrections, such as tests of normality and adjustment for multiple comparisons
☒	☐	A full description of the statistical parameters including central tendency (e.g. means) or other basic estimates (e.g. regression coefficient) AND variation (e.g. standard deviation) or associated estimates of uncertainty (e.g. confidence intervals)
☒	☐	For null hypothesis testing, the test statistic (e.g. *F*, *t*, *r*) with confidence intervals, effect sizes, degrees of freedom and *P* value noted<br>*Give P values as exact values whenever suitable.*
☒	☐	For Bayesian analysis, information on the choice of priors and Markov chain Monte Carlo settings
☒	☐	For hierarchical and complex designs, identification of the appropriate level for tests and full reporting of outcomes
☒	☐	Estimates of effect sizes (e.g. Cohen's *d*, Pearson's *r*), indicating how they were calculated

Our web collection on statistics for biologists contains articles on many of the points above.

Software and code

Policy information about availability of computer code

Data collection	Custom code and associated data files used for the genome analysis are available in the repository https://github.com/LeifAnderssonLab/Chen_et_al_2021_TSHR_functional, DOI: 10.5281/zenodo.4733728.
Data analysis	Custom code and associated data files used for the genome analysis are available in the repository https://github.com/LeifAnderssonLab/Chen_et_al_2021_TSHR_functional, DOI: 10.5281/zenodo.4733728.

For manuscripts utilizing custom algorithms or software that are central to the research but not yet described in published literature, software must be made available to editors and reviewers. We strongly encourage code deposition in a community repository (e.g. GitHub). See the Nature Research guidelines for submitting code & software for further information.

Data

Policy information about availability of data

All manuscripts must include a data availability statement. This statement should provide the following information, where applicable:

- Accession codes, unique identifiers, or web links for publicly available datasets
- A list of figures that have associated raw data
- A description of any restrictions on data availability

The raw ATAC-seq reads have been deposited in the NCBI Sequence Read Archive (SRA) under BioProject accession no. PRJNA660525.

Field-specific reporting

Please select the one below that is the best fit for your research. If you are not sure, read the appropriate sections before making your selection.

- [ ] Life sciences
- [ ] Behavioural & social sciences
- [x] Ecological, evolutionary & environmental sciences

For a reference copy of the document with all sections, see nature.com/documents/nr-reporting-summary-flat.pdf

Ecological, evolutionary & environmental sciences study design

All studies must disclose on these points even when the disclosure is negative.

Study description	The underlying molecular mechanisms that determine long day versus short day breeders are poorly understood. Atlantic herring provides a unique opportunity to examine the molecular mechanisms involved in reproduction timing, because both spring and autumn spawners exist within the same species. Although our previous whole genome comparisons revealed a strong association of TSHR alleles with spawning seasons, the functional consequences of these variants remain unknown. Here we examined the functional significance of six candidate TSHR mutations strongly associated with herring reproductive seasonality using various methods, including haplotype, open chromatin and sequence conservation analysis as well as the effects of two missense mutations on TSHR-cAMP signaling and modeled structures. Our study indicates that both coding and non-coding variants at the TSHR locus may contribute to the regulation of seasonal reproduction in herring.
Research sample	Tissue samples for expression analysis were collected from a population of spring-spawning herring kept in captivity at University of Bergen, and samples used for ATAC-sequencing were collected at Hästskär from wild spring-spawning Atlantic herring in the Baltic Sea.
Sampling strategy	N/A
Data collection	N/A
Timing and spatial scale	N/A
Data exclusions	N/A
Reproducibility	All assays in this study were performed at least twice as two independent experiments.
Randomization	N/A
Blinding	N/A

Did the study involve field work? [ ] Yes [x] No

Reporting for specific materials, systems and methods

We require information from authors about some types of materials, experimental systems and methods used in many studies. Here, indicate whether each material, system or method listed is relevant to your study. If you are not sure if a list item applies to your research, read the appropriate section before selecting a response.

Materials & experimental systems

n/a	Involved in the study	
[ ]	[x]	Antibodies
[ ]	[x]	Eukaryotic cell lines
[x]	[ ]	Palaeontology and archaeology
[ ]	[x]	Animals and other organisms
[x]	[ ]	Human research participants
[x]	[ ]	Clinical data
[x]	[ ]	Dual use research of concern

Methods

n/a	Involved in the study	
[x]	[ ]	ChIP-seq
[ ]	[x]	Flow cytometry
[x]	[ ]	MRI-based neuroimaging

Antibodies

Antibodies used	We used two custom antibodies in this study. One was used to confirm the production of recombinant herring scTSH, and the other one was used for the cell surface expression assay.
Validation	All antibodies were used and validated by our collaborators previously.

Eukaryotic cell lines

Policy information about cell lines

Cell line source(s)	All the cell lines used in this study were purchased from atcc.org
Authentication	Cells used in the study showed normal morphology and were in a healthy state.
Mycoplasma contamination	Cells used in the study were not contaminated with mycoplasma
Commonly misidentified lines (See ICLAC register)	N/A

Animals and other organisms

Policy information about studies involving animals; ARRIVE guidelines recommended for reporting animal research

Laboratory animals	This study did not involve laboratory animals
Wild animals	Tissue samples used for ATAC-sequencing were collected at Hästskär from wild spring-spawning Atlantic herring in the Baltic Sea.
Field-collected samples	This study did not involve samples collected from field
Ethics oversight	Rearing of the captive herring was approved by the Norwegian national animal ethics committee (Forsøksdyrutvalget FOTS ID-5072).

Note that full information on the approval of the study protocol must also be provided in the manuscript.

Flow Cytometry

Plots

Confirm that:

- [x] The axis labels state the marker and fluorochrome used (e.g. CD4-FITC).
- [x] The axis scales are clearly visible. Include numbers along axes only for bottom left plot of group (a 'group' is an analysis of identical markers).
- [x] All plots are contour plots with outliers or pseudocolor plots.
- [x] A numerical value for number of cells or percentage (with statistics) is provided.

Methodology

Sample preparation	Cells expressing TSHR were harvested 24 h after transfection, then washed once in cold PBS and fixed in 2% PFA for 10 min at room temperature, which were used for the subsequent flow cytometry analysis.
Instrument	CytoFLEX instrument (Beckman Coulter)
Software	CytExpert
Cell population abundance	Cells were gated based on the FSC-A and FITC-A. Two negative controls were used in this analysis, one included the cells without any antibody stainings, the other one included cells transfected with empty pcDNA3.1 vector.
Gating strategy	Cells were first gated based on the FSC-A and FSC-W to identify the single cells, then gated based on the FSC-A and FITC-A to identify the positively stained cells.

- [x] Tick this box to confirm that a figure exemplifying the gating strategy is provided in the Supplementary Information.
